# Intranodal palisaded myofibroblastoma originating from retroperitoneum: an unusual origin

**DOI:** 10.1186/1472-6890-11-7

**Published:** 2011-06-30

**Authors:** Jayesh Sagar, Athanasia Vargiamidou, Hemachandran Manikkapurath

**Affiliations:** 1 Department of Surgery, Royal Sussex County Hospital, Brighton. BN2 5BE, UK; 2 Department of Cellular Pathology, Stoke Mandeville Hospital, Aylesbury. HP21 8AL, UK

**Keywords:** Intranodal palisaded myofibroblastoma, retroperitoneum, lymph nodes

## Abstract

**Background:**

Intranodal palisaded myofibroblastoma is one of the primary mesenchymal tumours. The inguinal region is the commonest site of this rare tumour. As there are only about 55 such cases reported in the literature, the precise aetiology and pathogenesis have yet to be explained adequately. Here we report a case of a 72 year old man presented with incidental finding of intranodal palisaded myofibroblastoma in the retroperitoneal region.

**Case Presentation:**

A 72-year old man presented with abdominal pain in right upper quadrant with an incidental finding of abdominal mass in the right flank. The computerised tomogram scan of abdomen confirmed acute cholecystitis with a 5 x 5 cm retroperitoneal mass. He underwent cholecystectomy with excision of this mass. He recovered well following his operation and was discharged from the hospital. Histological examination confirmed the diagnosis of intranodal palisaded myofibroblastoma.

**Conclusion:**

To our knowledge, this is the first case of intranodal palisaded myofibroblastoma originating from retroperitoneum. Along with the rarity of this case, we also discussed its typical histopathological findings, aetiology and pathogenesis.

## Background

In comparison to secondary mesenchymal tumours, primary mesenchymal tumours involving lymph nodes are very rare. The most common primary mesenchymal tumours include Kaposi's sarcoma, pseudosarcomatous malignant melanomas and melanocytic naevi [1]. Intranodal palisaded myofibroblastoma (IPM), one of the rare primary mesenchymal tumours, was first described in English literature in 1968, though not classified as Intranodal palisaded myofibroblastoma [2]. These tumours arise from the lymph nodes and are almost always seen in the inguinal lymph nodes, although few cases of mediastinal and submandibular lymph node origins have also been reported [3]. Its unique microscopic, macroscopic and immunohistochemical features differentiate it from other mesenchymal tumours. IPM shows both myofibroblastic and smooth muscle differentiation with the formation of the "amianthoid fibres". To date only about 55 cases of IPM have been reported in the English literature [4-6]. In this case report we presented another case of IPM, but originating from retroperitoneum. To our knowledge, this origin of IPM has not yet been reported. Apart from the rarity of this tumour, we also discussed its characteristic features, aetiology and pathogenesis.

## Case Presentation

A 72 year-old man presented in the emergency department with right upper quadrant and epigastric pain, nausea and vomiting. On examination, the patient was sweating and pale with a tender right upper quadrant. A tender 5 × 5 cm^2 ^mass was also noted in the right flank. The provisional diagnosis of acute cholecystitis with incidental finding of abdominal mass was made and further investigation in the form of computerised tomography (CT) of his abdomen and pelvis was performed. The scan confirmed the presence of 50 × 55 mm^2 ^mass in the retroperitoneum on the right side of the urinary bladder but separate from the small intestinal loops without any evidence of local infiltration. The radiological differential diagnosis included carcinoid lesion and teratoma of a right undescended testis. However, undescended testis was ruled out with a fully grown testis on the right side of the scrotum. The patient underwent exploratory laparotomy, open cholecystectomy and also excision of the pelvic mass. Intra-operatively, the mass was found to be approximately 8 cms in size, partly haemorrhagic and covered by peritoneum. It was situated lateral to the right ureter and superior to the right iliac vessels. This mass was excised en-block without any intra-operative complications. The patient had an uneventful post-operative recovery and was discharged on the 10th post-operative day. Histological diagnosis of intranodal palisaded myofibroblastoma was confirmed. He did not reveal any signs of recurrence in seven years of follow up. The histopathological findings are described as follows.

### Macroscopic characteristics

A Solid mass measuring 80 × 80 × 50 mm^3 ^with surrounding attached fatty tissue measuring up to 100 mms in maximum dimension was noted. On slicing, a well defined nodule with partially firm, white and solid areas, alternating with haemorrhagic areas, was found. At the periphery there was some calcification. The microscopic study was performed on extensively sampled solid and haemorrhagic areas including the resection margins. The gall bladder was 105 mms with a thick wall and a few tiny stones.

### Microscopic description

**Figure 1:**
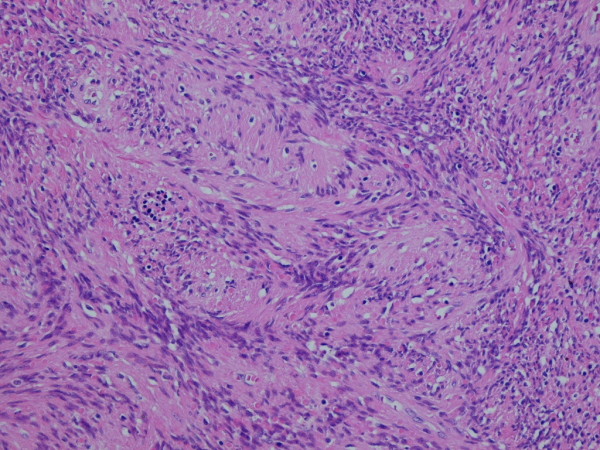
**High power view of the tumour with interlacing fascicles of spindle cells**. H & E × 10 objective.

**Figure 2:**
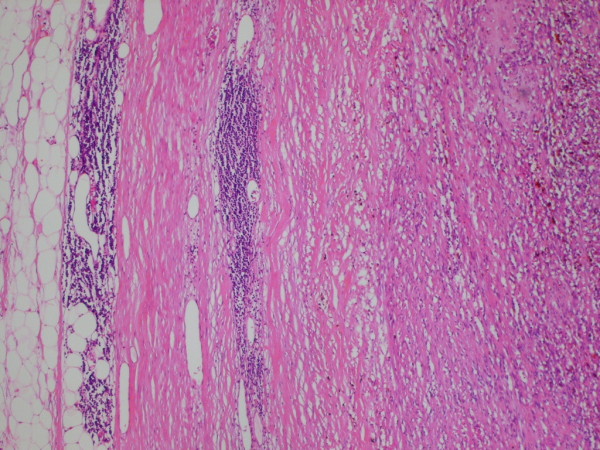
**Low power view of the tumour with the capsule and lymphoid cell infiltrate at the periphery**. H & E × 4 objective.

**Figure 3:**
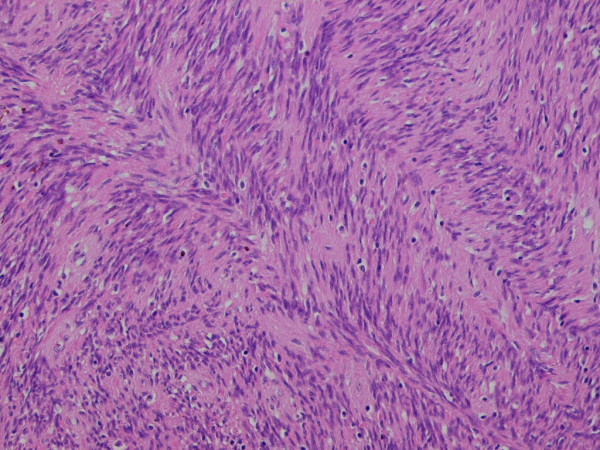
**High power view of the tumour showing prominent fascicular growth pattern with many short and long interlacing fascicles**. H & E × 20 objective.

**Figure 4:**
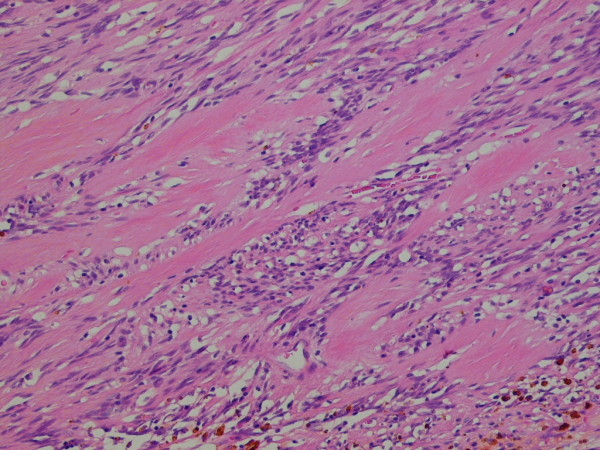
**High power view of the tumour showing amianthoid fibres**. H & E × 40 objective

This represented encapsulated, a well defined nodular lesion with variable cellularity. The cells were spindle shaped with slightly wavy nuclei forming short interlacing fascicles, exhibiting areas that were reminiscent of AntonyA/Antony B area (figure 1). No significant nuclear pleomorphism was visible however mitosis was rarely noted in 1 in up to 30 high power field (× 40 objective). Areas of fresh as well as old haemorrhages were present. At the periphery, reactive lymphoid infiltrate and focal calcification were also noted (figure 2). The tumour showed a prominent fascicular growth pattern with many short and long interlacing fascicles of tumour cells (figure 3). Amianthoid fibres were easily identified though in few areas of the tumour (figure 4).

### Immunohistochemical Staining

**Figure 5:**
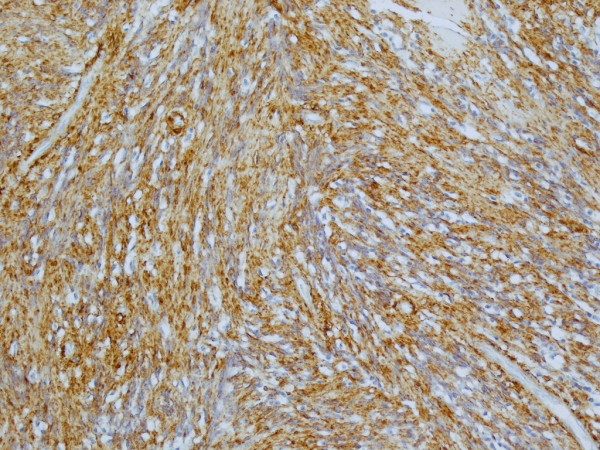
**High power view of the tumour showing strong expression of smooth muscle marker, SMA**. × 10 objective

**Figure 6:**
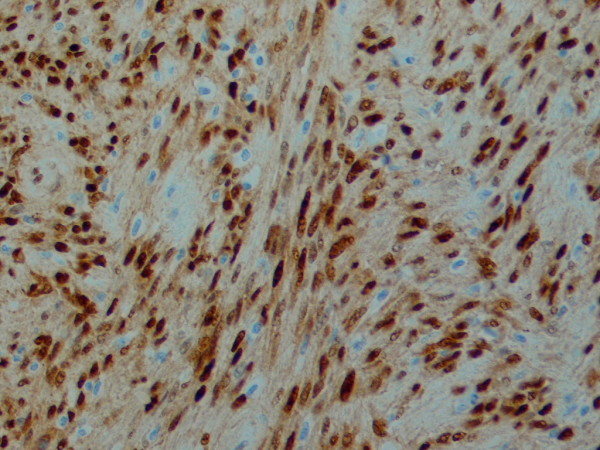
**High power view of the tumour cells showing strong nuclear expression of Cycline D1**. × 40 objective

**Figure 7:**
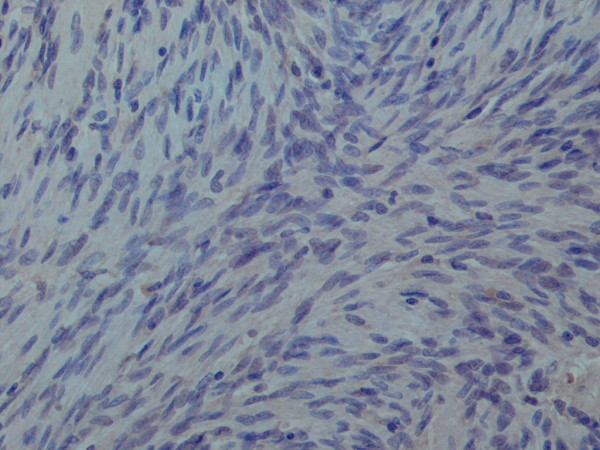
**High power view of the tumour cells showing negative expression Alk-1**. × 40 objective.

**Figure 8:**
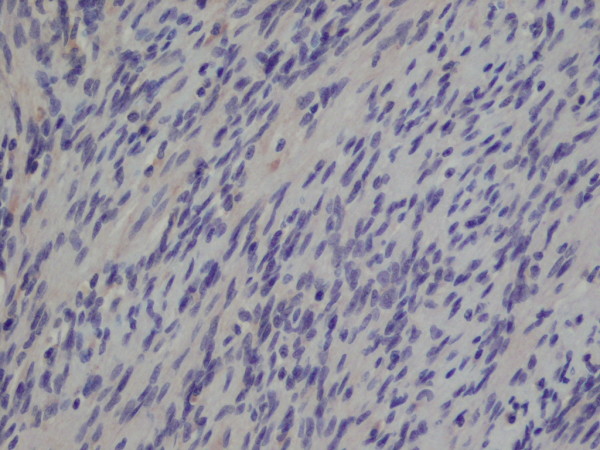
**High power view of the tumour cells showing negative expression of HHV 8**. × 40 objective

The spindle cells were positive for Smooth muscle actin (SMA) (Figure 5), Calponin and Vimentin but did not express Desmin and h-Caldesmon. The tumour cells showed strong nuclear expression of Cyclin D1 (figure 6). The cells were negative for neurofilament, cytokeratin. The staining for S100 protein, CD34, CD99, Bcl-2 and CD117/C-kit were negative in the tumour cells. Immunostaining for Alk-1 and HHV 8 were negative in these cells (figure 7 & 8).

## Conclusions

Among primary mesenchymal tumours, intranodal palisaded myofibroblastoma is very rare. It was initially described by Deligdish and Katz as neurilemmoma or schwanoma and later classified as palisaded myofibroblastoma by Weiss [7,8]. It shares many features of myofibroblasts and smooth muscle cells microscopically as well as immunohistochemically. This mesenchymal spindle cell tumour arises from the lymph nodes. It is most commonly seen in the inguinal lymph nodes, however origins from submandibular and mediastinal lymph nodes have also been reported [3].

IPM commonly affects the second to eighth decade, with a peak incidence in the group between 40 and 60 years of age, but occurrence of this tumour in an infant has also been reported [9,10]. It is more common in men with a male to female ratio 1.5:1 and is not specific to any race [11]. The most common clinical presentation is a painless slow growing lump in the inguinal region. With increasing size discomfort, pain and compression of local surrounding structures may manifest various symptoms. It is most often diagnosed clinically as lymphadenopathy and histological diagnosis is established on excision biopsy. Fortunately, this tumour is associated with very low recurrence rate and the recurrence is almost benign in nature. In a series of 30 cases, only 6% was reported to demonstrate benign local recurrence [12,13]. Creager et al reported only a single case of intranodal palisaded myofibroblastoma with metastatic bone formation [13].

It is essential to differentiate IPM from other soft tissue tumours. These include Schwannoma, Kaposi's sarcoma, intranodal leiomyoma, inflammatory pseudotumour, solitary fibromastocytic tumour, angiomyomatous hamartoma and metastatic spindle cell lesions in the lymph node. The clinical history, examination and typical histological characteristics help in the correct diagnosis of the IPM. Schwannoma is uncommon in the inguinal region and immunohistochemically it is positive for S100 that differentiates it from IPM. Negative immunostaining for HHV 8 along with an absence of EBV DNA and a history of immunocompromised status favours the diagnosis of IPM rather than Kaposi's sarcoma. There were no slit-like vascular channels, no extravasation of red blood cells and no hyaline globules as typically seen in Kaposi's sarcoma. Differentiation of inflammatory myofibroblastic tumour (IMT) from IPM can be made by the presence of inflammatory cells and absence of the amianthoid fibers in IMT [14]. Negative immunostaining for Alk-1 and the presence of amanthoid fibres with absence of inflammatory cell infiltrate, including lymphocytes and plasma cells, favoured the diagnosis of IPM. Furthermore, spindle cell melanoma can be differentiated from IPM by high proliferative activity and marked cellular atypia. Spindle cell melanoma is also positive for S100 and HMB-45 [15]. The gastrointestinal stromal tumour is positive for CD117/C-kit while IPM is negative. Regarding angiomyomatous hamartomas in lymph nodes, the morphology and immunostaining patterns are consistent with IPM rather than angiomyomatous hamartomas.

Due to the rarity of the condition the precise aetiology and pathogenesis have yet to be defined. Inguinal lymph nodes have a higher concentration of myofibroblasts compare to other lymph nodes in the body due to increased drainage area and function: this might explain its more common occurrence in the inguinal region. This is favoured by positive staining for actin and vinmentin and negative for desmin in myofibroblasts of the inguinal region compared to fibroblasts elsewhere in the body [2]. However, its appearance in other parts of the body contradicts this hypothesis. As in our case, the origin from the retroperitoneal region has not been reported yet in literature and this again raises the question of the aetiology of IPM.. The positive staining for cyclin D1 suggests the possible role of the cell cycle regulatory genes in the pathogenesis of IPM [16]; thus reporting of more number of cases is required to get a better understanding of the aetiology and pathogenesis of IPM.

Although rare, IPM should be considered as one of the differential diagnosis in patients presenting with an abdominal mass in the retroperitoneal region. This site of origin may provide an opportunity to reconsider the aetiology and pathogenesis of IPM.

## Abbreviations

IMT: Inflammtory Myofibroblastic Tumour, IPM: Intranodal Palisaded Myofibroblastoma

## Consent

Written informed consent was obtained from the patient for publication of this case report and accompanying images. A copy of the written consent is available for review by the Editor-in-Chief of this journal.

## Competing interests

The authors declare that they have no competing interests.

## Authors' contributions

JS was involved directly in patient management and preparation of manuscript. AV was involved in preparation of manuscript. HM provided the histological pictures with picture legends. All authors read and approved the final manuscript.

## Pre-publication history

The pre-publication history for this paper can be accessed here:

http://www.biomedcentral.com/1472-6890/11/7/prepub
